# Access to Dental Care for Children and Young People in Care and Care Leavers: A Global Scoping Review

**DOI:** 10.3390/dj12020037

**Published:** 2024-02-08

**Authors:** Jo Erwin, Jane Horrell, Hannah Wheat, Nick Axford, Lorna Burns, Joelle Booth, Robert Witton, Jill Shawe, Janine Doughty, Sarah Kaddour, Skye Boswell, Urshla Devalia, Abigail Nelder, Martha Paisi

**Affiliations:** 1 Peninsula Dental School, University of Plymouth, Drake Circus, Plymouth PL4 8AA, UK; lorna.burns@plymouth.ac.uk (L.B.); joelle.booth@plymouth.ac.uk (J.B.); robert.witton@plymouth.ac.uk (R.W.); martha.paisi@plymouth.ac.uk (M.P.); 2 Peninsula Medical School, University of Plymouth, Drake Circus, Plymouth PL4 8AA, UK; jane.horrell@plymouth.ac.uk (J.H.); hannah.wheat-1@plymouth.ac.uk (H.W.); nick.axford@plymouth.ac.uk (N.A.); 3 Centre for Dental Public Health and Primary Care, Queen Mary University of London, Turner Street, London E1 2AD, UK; 4 Peninsula Dental Social Enterprise, Plymouth PL6 8BT, UK; abigail.nelder@plymouth.ac.uk; 5 School of Nursing and Midwifery, University of Plymouth, Drake Circus, Plymouth PL4 8AA, UK; jill.shawe@plymouth.ac.uk; 6 School of Dentistry, Royal Liverpool University Dental Hospital, Pembroke Place, Liverpool L3 5PS, UK; janineyd@liverpool.ac.uk; 7 Pathway Oral Health Fellow, Pathway, 250 Euston Road, London NW1 2PG, UK; s.kaddour@nhs.net; 8 Patient and Public Involvement Member, Plymouth County Council, Plymouth PL1 3BJ, UK; 9 Royal National ENT and Eastman Dental Hospitals, University College London Hospitals, London NW1 2BU, UK; urshla.devalia@nhs.net

**Keywords:** global oral health, access, dental care, children’s oral health, children looked after, orphans, vulnerable children, unaccompanied refugee asylum minors, foster

## Abstract

Aims: This scoping review aimed to explore three research questions: 1. What is the dental care access for children and young people (CYP) in care and care leavers? 2. What factors influence CYP in care and care leavers’ access to dental care? 3. What pathways have been developed to improve access to oral health care for CYP in care and care leavers? Methods: Five databases (Ovid MEDLINE, Ovid Embase, CINAHL, SocINDEX and Dentistry and Oral Sciences Source) and grey literature sources were systematically searched. Articles relating to CYP in care or care leavers aged 0–25 years old, published up to January 2023 were included. Abstracts, posters and publications not in the English language were excluded. The data relating to dental care access were analysed using thematic analysis. Results: The search identified 942 articles, of which 247 were excluded as duplicates. A review of the titles and abstracts yielded 149 studies. Thirty-eight were eligible for inclusion in the review: thirty-three peer-reviewed articles, one PhD thesis and four grey literature sources. All papers were published from very high or medium Human Development Index countries. The studies indicate that despite having higher treatment needs, CYP in care and care leavers experience greater difficulty in accessing dental services than those not care-experienced. Organisational, psycho-social and logistical factors influence their access to dental care. Their experience of dental care may be impacted by adverse childhood events. Pathways to dental care have been developed, but little is known of their impact on access. There are very few studies that include care leavers. The voices of care-experienced CYP are missing from dental access research. Conclusions: care-experienced CYP are disadvantaged in their access to dental care, and there are significant barriers to their treatment needs being met.

## 1. Introduction

Half of the world’s population suffers from oral diseases, with three out of every four affected people living in low- and middle-income countries [1]. Untreated caries in deciduous teeth is the single most common chronic childhood disease, affecting an estimated 514 million children worldwide [2]. The inequalities in access to oral health services are stark, with a huge burden of oral diseases and conditions affecting the most vulnerable and disadvantaged populations [1]. 

Although poor oral health is largely preventable, due to the complex interplay of a range of factors globally, there are some population groups that experience worse oral health than their peers. One of these groups is children in care and care leavers. Children and young people (CYP) entering care are more likely to have a history of poor oral health than their peers, which may arise from challenging life circumstances such as poverty, neglect and abuse [3,4,5,6,7,8,9,10]. After entering care, they continue to experience poorer oral health than their peers, including dental decay [11,12], periodontal disease [13,14] and dental trauma [15,16]. Upon leaving care, they have less access to dental care [17]. 

Poor oral health and untreated dental disease can significantly impact CYP’s general health and well-being. They can experience pain, discomfort, loss of sleep, stigmatisation and bullying, which impacts their self-confidence, quality of life and school performance [18,19,20,21]. The impact of poor oral health can further contribute to disadvantage and inequality. In contrast to other chronic diseases, teeth are a strong marker of social class and provoke value judgements such as perceptions of lower relational competency and educational level [22]. 

The concept of access to health services is complex and can be defined in a number of ways [23]. At its most fundamental, access depends on the existence of available services in adequate supply, the population’s ability to utilise the service (which may be compromised by cost, organisational, social and cultural factors) and the ability of the service to offer effective health outcomes [24].

Having access to effective dental care is an important aspect of maintaining good oral health. Both children in care and care leavers experience greater difficulty in accessing dental services [3,25,26,27,28,29,30] and are less likely to access dental services compared to other children in the general population [31,32,33,34]. After leaving care, CYP continue to have compromised access to dental care [17,35]. Research from the Global South also suggests that children and young people in care are disadvantaged in accessing oral health care and receiving treatment [15,36,37,38,39]. There has been little research exploring this inequality [40]. 

The UK’s National Institute for Health and Clinical Excellence (NICE) Public Health Guidelines on children and young people in care has raised concerns about access to dental care for children in care and care leavers [41]. Identifying and understanding the factors influencing access to dental care by children in care and care leavers can help inform recommendations for effective programmes and services that respond to the oral health needs of these children and young people. 

A recent scoping review by Hurry et al. [40] examined the evidence relating to dental access for CYP in care and care leavers in the UK. At present, there are no reviews collating the global evidence on the factors that influence access to dental care by CYP in care and by care leavers. This scoping review aims to help fill this evidence gap by systematically searching the literature to identify and describe the global evidence on access to dental care for this population. It addresses the following three research questions: 

RQ1: What is the dental care access of CYP in care and care leavers? 

RQ2: What factors influence CYP in care and care leavers’ access to dental care? 

RQ3: What pathways have been developed to improve access to oral health care for CYP in care and care leavers?

## 2. Methods

The evidence presented here represents part of a wider scoping review that not only looked at access to dental care but also at the oral health status of children in care and care leavers, their oral health behaviours and interventions developed for oral health promotion and/or care. A protocol for this scoping review was developed a priori. 

### 2.1. Inclusion and Exclusion Criteria

This review focused on children and adolescents removed from biological parents and residing in informal foster care, formal foster care or residential care. Individual studies were included in this review if they (i) reported on children and adolescents aged 18 years or younger who are currently in formal/informal foster or residential care and/or care leavers aged up to 25 years; (ii) pertained to dental care access/provision; and (iii) were published in the English language. No restrictions were placed on the country or date of publication. Studies were excluded if they (i) reported on individuals over the age of 25 years; (ii) focused exclusively on dietary data; and (iii) were not written in the English language; (iv) were published as only abstracts or posters or were unpublished work. 

### 2.2. Search Strategy 

An experienced information specialist (L.B.) conducted a systematic search of the literature related to children and adolescents in care and dental care access/provision The initial search was conducted on 3 October 2022 and updated on 8 January 2023 and 2 January 2024. Five electronic databases were searched: Ovid Embase, Ovid MEDLINE, CINAHL (EBSCOhost), SocINDEX (EBSCOhost) and Dentistry and Oral Sciences Source (EBSCOhost). Grey literature sources included Google, EThOS, the Health Foundation, Social Care Online, ClinicalTrials.gov, Fostering Network Voice of the Child in Care, NSPCC and Who Cares Trust, Safeguarding Network, Early Intervention Foundation, Barnardo’s. Search strategies are shown in Appendix A.

The database searches comprised both subject headings and title abstract terms for the concepts of children in foster or residential care and oral health. The search terms were attained via scoping searches and via discussions with the research team and stakeholders. Forward and backward citation searches on the included studies were conducted to supplement the search. No date or country limits were applied. 

### 2.3. Study Records

#### 2.3.1. Data Management/Selection Process

Search results were exported to Endnote 20 (Clarivate Analytics (US) LLC), and duplicates were removed. Subsequently, the records were exported to Rayyan [42]. Two independent reviewers (J.H. and J.E.) screened all titles, abstracts and full texts against the eligibility criteria. Any disagreements regarding inclusion were settled via discussion between the two reviewers. Where consensus could not be reached, a third reviewer (M.P.) was consulted.

#### 2.3.2. Data Extraction

A data extraction sheet was created, and the independent reviewers extracted the data using this proforma. The data extraction sheet included the following information: author(s) and year of publication; aim and objectives; study design; duration country with associated HDI [43]; sampling method; sample size; participant characteristics (age, gender, ethnicity and socioeconomic status); other participant characteristics (e.g., type of care); setting; description of intervention (if applicable); data collection tools; dental care access/provision; results; conclusions and recommendations/gaps in research.

#### 2.3.3. Data Synthesis

The synthesis of the data was informed using the Arksey and O’Malley framework [44]. This included identifying the research question; identifying relevant studies; study selection; charting the data; and collating, summarising and reporting the results. The results were summarised to present an overview of the evidence. Quantitative and qualitative analyses were used to describe study characteristics. This enabled major themes to be identified and refined (J.E., J.H., and H.W.) and for gaps in the literature to be identified. 

### 2.4. Patient and Public Involvement (PPI)

PPI group and stakeholder representatives were involved in writing and refining the protocol of the review, interpreting the results and contributing to the manuscript. The PPI group is made up of young people currently in care and care leavers who are actively working with the research team. 

## 3. Results 

**Figure 1:**
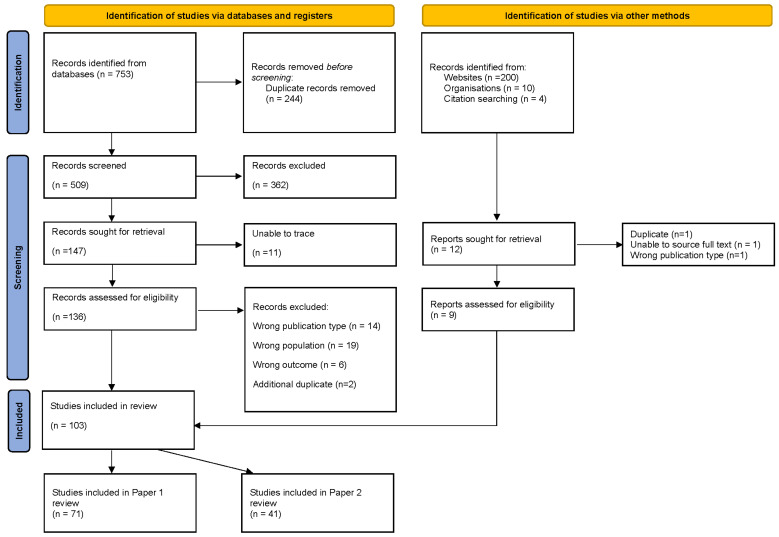
PRISMA flowchart.

Figure 1 depicts the flow of information through the different phases of the scoping review. The initial search identified 753 articles. After the removal of duplicates, 509 remained. Title and abstract screening led to a further 362 records being excluded. Of the remaining 147 articles, 12 were unable to be traced, and 41 were excluded for the following reasons: wrong publication type (*n* = 14), wrong population (*n* = 19) or wrong outcome (*n* = 6). The total number of articles included in the review was 103. 

### 3.1. Study Characteristics 

The studies identified relating to access to dental care totalled 41:36 peer-reviewed articles, 1 PhD thesis and 4 grey literature sources.

Thirty-eight of the sources were from very high HDI countries, and three came from India, a medium HDI country. The majority of sources were from the USA (32%), UK (23%) and Australia (17%). Other countries included Egypt, Sweden, Malta, Canada and Germany. The published study designs included cross-sectional, mixed method, qualitative and retrospective cohort studies, service evaluation and a systematic review. The ages of the CYP participating in the studies ranged from 0 to 29 years. Residency types varied across the studies from orphanages, out-of-home care, foster care, residential care homes and kinship care. 

### 3.2. RQ1: What Is the Dental Access of Children and Young People in Care and Care Leavers? 

Eleven studies referenced access to dental services. These came from very high (*n* = 10) or medium (*n* = 1) Human Development Index (HDI) countries. They reported that CYP in care were less likely to visit the dentist regularly [9,34,45,46,47,48] or visit the dentist in line with recall guidance [49] and were more likely to require treatment when they did attend a dental practice [31]. CYP with decay were more likely to have visited the dentist, whereas CYP who reported dental pain were less likely to have attended in the past year [50]. In the UK, children entering care had higher treatment needs than their peers [51]; some had little or no experience of attending the dentist before entering care [3,52]. Thus, they did not have the opportunity to acclimatise that their peers did. Once in care, CYP continue to have poor attendance [3,34,53]. A Scottish study found that CYP in care were half as likely as those not in care to regularly attend dental services [34]. In Canada, CYP in care had significantly higher rates of visits to (non-dental) physicians for oral health needs than CYP not in care [54]. 

The American Association of Paediatric Dentistry suggest that all children receive a preventative dental visit every 6 months [55]. Melbye et al. [56] found that in the US state of Washington in 2008, only 43% of fostered children received any dental care and that this level of care was similar to that which was received by all publicly insured children. Melbye suggests that this is because they experience the same barriers to accessing dental care. In Scotland, CYP in care had double the rate of urgent treatment and were twice as likely to have required extractions under general anaesthetic [34]. 

In Sweden, regardless of time spent in care, care leavers were less likely to have received check-ups and more likely than those not care-experienced to have made emergency visits and undergone extractions; this differential increased with age [57]. 

### 3.3. RQ2: What Factors Influence Care-Experienced Children and Young People’s Access to Dental Care? 

Twenty-eight sources contributed to research question two. Of these, twenty-three were peer-reviewed papers, one was a PhD thesis and four were grey literature. All originated from very high HDI countries except one [58], which was from India, a medium HDI country. Of the peer-reviewed sources, eleven were studies which were cross-sectional in design. Eleven drew on retrospective dental charts, register reviews or service level data. Thirty percent used qualitative methods. Four studies from Malta, the UK and Australia included carers [3,49,52,59], two from the US and UK included social workers [3,6] and two from the UK included dental health professionals [3,53]. Fourteen studies used data relating to CYP in care, and two studies from the UK and Sweden included data from care leavers [17,57]. 

The themes identified relating to the accessibility of dental care are organisational factors, psycho-social factors, location of services, financial factors and factors affecting the dental care experience.

### 3.4. Organisational Factors Affecting Access to Dental Care

#### 3.4.1. Carers and Health/Social Care Professionals Lack of Knowledge of the Process for Obtaining Access to Dental Care for the Children and Young People in Their Care

Carers’ uncertainty about the processes to secure dental care is a barrier to CYP’s access to care [52]. Carers experience difficulties in accessing care, whether via referral agencies (foster care agencies) [7] or, in the UK, the general dental service (GDS) [3]. In Australia, even when approval from foster care agencies had been granted, carers reported experiencing long waits to achieve access [7]. In England and Wales, Community Dental Service professionals’ knowledge of services available to CYP in care is variable [53]. A UK study [52] of access to dental care for very young children found that carers experienced difficulty in accessing the dental care the children should receive as specified by statutory guidance. This was because dental professionals were unaware of this guidance. This difficulty in access created a barrier to carers’ future engagement with dental health professionals.

A US study identified that while social workers felt confident in finding a dentist for CYP in care in an emergency, they were less confident in locating one to provide routine care [6]. 

#### 3.4.2. Insufficient Sharing of CYP’s Oral Health Information between Foster Carers, Health and Social Care Professionals

Social workers in the US [6] reported difficulties in obtaining dental histories for children entering foster care. A common reason for not having up-to-date dental records was children “bouncing” from one foster home to the next. In a study from Australia, foster carers remarked on the lack of information provided by fostering agencies on children’s oral health and dental care needs. This made planning for the child’s oral health care more difficult for the carers [7]. A lack of information on the CYP’s dental history can be a challenge to the provision of dental care, particularly where CYP are accompanied at dental appointments by people (e.g., transporters and new foster parents) who may have little or no knowledge of the child’s medical or social history [60]. A survey of public health nurses (PHN) in California, where Health and Education Passports (HEP) are mandatory for children in foster care, found that the HEP were mostly used by social workers, PHNs and mental health providers [61]. The HEP were used at medical and dental visits and played an important role in bringing together the health history of the child. 

#### 3.4.3. CYP in Care and Care Leavers’ Changes in Location and Duration of Placement 

A US study [62] found that being placed in care increased the likelihood of accessing dental care, with more days in placement being associated with a greater likelihood of receiving dental care. One explanation for this is the requirement for an intake health assessment, which may prompt access to dental services. The association with the number of days in placement may also be explained by the increased oversight of oral health by social workers and/or carers [63]. 

A high frequency of placement moves and shorter placements can negatively impact on CYP’s ability to access dental care [41]. In the US, the utilisation of dental care was found to be less common in children who were enrolled in foster care for only part of the year [64], and CYP without a usual source of dental care had significantly lower chances of visiting the dentist within the previous year than those with a usual dentist [46]. This may be of particular relevance to older CYP as they tend to experience higher numbers of placements [7,65]. Short-term placements may also result in carers being more focused on more urgent mental health issues rather than prioritising dental health [7]. 

#### 3.4.4. Social Worker and Health Professionals’ Role in Facilitating Access to Dental Care 

Children in foster care are often dependent on social workers to protect their health and well-being [6,17]. In the UK, the local authority has responsibility for meeting the needs of the CYP in their care, including dental care, usually via a named social worker. Carrellas [17] emphasises that social workers should ensure that the CYP in their care are accessing preventative oral health care and dental treatment and that social workers should promote increased access to and utilisation of these services by CYP in care. Negro et al. [6] evaluated the feasibility of social worker-delivered oral health interventions for preschoolers in foster care. Social workers considered themselves to be best placed to lead brief oral health programmes during home visits. They recognised they would require further education and training to fulfil this role. Social workers play an important role in working with foster carers to facilitate routine dental care. However, this provision may be compromised by heavy caseloads and frequent staff changes [65]. Also, organisations vary in the value placed on social workers’ relationship-building with carers and CYP, which may affect carers’ and CYP’s ability to access dental care [65]. 

In India, CYP in orphanages are usually taken care of by staff from non-governmental organisations (NGOs) or social workers; Khare [36] recorded the poor oral health and access to dental care of these CYP and suggests that this is in part due to the NGO and social workers failure to realise that dental care and oral health are an integral part of CYP’s health and well-being. 

Carabez et al. [66] describe the role of Public Health Nurses in the US in addressing the needs of children and adolescents in foster care. Their study demonstrated that public health nursing expertise is an essential part of the child welfare team in addressing the medical, dental, mental and developmental needs of children in foster care. In Scotland, a study investigated whether a specialist nursing service could improve the health care of children in residential care [67]. It found that after the introduction of this service, the number of children registered with a dentist increased from 14 percent to 62 percent and the proportion of children with completed carer-held health records from three percent to 77 percent. In the more urban areas covered by the service, the main advantage of the service was considered to be in the facilitation of interagency working.

### 3.5. Psycho/Social Factors

#### 3.5.1. Type of Placement

Children in kinship care in the USA were found to have health problems similar to those in foster and poor children but more problems than American children in general [68]. Among the frequent diagnoses was dental caries, which often went untreated. 

#### 3.5.2. Dental Neglect

Dental neglect is defined as a type of child neglect [69,70,71], which includes parents’ or carers’ failure to access the necessary treatment needed to maintain their child’s oral health when dental services are available. A UK study examined the prevalence of two types of dental neglect in adolescents attending school in a deprived area [16]: neglect of prevention of oral disease (DPN) and neglect of dental treatment (DTN). For both types of neglect, a higher proportion of CYP in care experienced dental neglect than their peers who were not in care; 52% of CYP in care had experienced dental caries (DPN) vs. 39% of CYP not in care, and 65% of CYP had at least one untreated dental condition and/or pain (DTN) vs. 41% of their peers not care-experienced. 

#### 3.5.3. Barriers to Foster Carers Facilitating Access to Dental Care 

Melbye et al. [65], as part of their study of the determinants of access to dental care by children in foster care, examined the role of foster carers. Cultural and language barriers to dental care access were identified. For some foster parents, where English was a second language, they would avoid communicating with dental staff. This impacted on access and completion of their child’s recommended dental treatment. It was also observed that some foster carers had “different cultural ideas” about whether regular dental care is important. Competing demands, for example, caring for a CYP with additional needs or caring for more than one child, also impacted negatively on access [65].

#### 3.5.4. CYP’s Negative Attitude toward Dental Care

A UK study [3] suggested that CYP demonstrating a negative attitude toward maintaining their oral health may act as a barrier to their accessing dental care. This is more evident in older CYP, who may be more likely to challenge the parental authority of their carer [52] or exercise their right to refuse care [65]. CYP may feel uncomfortable going to the dentist because they have previously missed or cancelled appointments because of factors outside of their personal control, for example, not being taken to the appointment or lack of transport [3]. 

Care-experienced CYP said they do not want to be singled out as “different” by attending a specialised service when they were already attending a multitude of specialist appointments due to being in care. Instead, care leavers suggested that the specialised training of dental professionals in the issues that may influence the oral health behaviours of care-experienced CYP would be of more benefit [9].

#### 3.5.5. CYP’s Anxiety about Visiting the Dentist

CYPs may find attending the dentist anxiety-inducing for a range of different reasons, including fear of the unknown, parental dental fear, experience of toothache and previous painful dental treatment [72]. This anxiety may be exacerbated for those CYP who may have had little or no experience of attending the dentist or receiving treatment before and/or after entering care [52]. In a qualitative study from the UK, it was found that care-experienced CYP had high levels of anxiety associated with fear of dental treatment [3]. CYP who were still registered with their biological family dentist experienced anxiety because of the potential risk of encountering their birth parents [3]. Anxieties may result in the CYP’s last-minute refusal to attend a booked appointment or to attend even if they are experiencing a toothache. This could lead to the child being refused further treatment by the provider [3]. Foster carers were identified by care leavers in the UK as playing a key role in supporting anxious CYP to attend their appointments, preparing them for what to expect at an appointment, and teaching them how to navigate the health service [9]. 

The one study not from a very high HDI country, India, found that CYP in care had less dental anxiety than their peers [58] as measured using the children’s fear survey schedule-dental subscale and modified faces version of the modified child dental anxiety scale. The authors, surprised by this observation, suggested that the guidance and counselling services offered to orphans perhaps helped them to understand their problems and deal more effectively with their emotions, including anxiety. 

### 3.6. Logistical and Financial Factors

#### 3.6.1. Geographical Location

The evidence on the impact of location is mixed. In one Australian study, rurality limited dental access due to restricted services, resulting in a proportionally low number of dental referrals compared to CYP in care living in metropolitan areas [73]. In contrast, two studies, one from Australia [74] and the other from the US [56], showed increased health service use in regional/rural areas. McClean et al. [74] hypothesised that this may be because systems are easier to navigate in locations with fewer options for healthcare and where CYP are likely already known to the local health services. An explanation suggested by Melbye [56] is that compared to those in metropolitan areas, dentists and rural dentists may experience an increased sense of social responsibility to accept Medicaid-enrolled children because community clinics may not be as readily available. 

#### 3.6.2. Distance to Travel 

NICE [41] identifies the need for CYP in care to travel substantial distances to see a dentist as a factor influencing access to dental care. Data from the UK [3], the US [8] and Australia [7] also indicate that the distance to travel can be a barrier to CYP in care and attending dental appointments. This may be a particular issue for CYP who have been placed in a different area but wish to continue care under their existing dentist [3]. 

#### 3.6.3. Cost and Availability of Medical Insurance 

Despite there being evidence that being in receipt of state assistance increases the likelihood of receiving at least one dental visit [75], limited insurance availability and restrictions (USA and Australia) can mean that carers find locating an approved dentist difficult or that insurance provision is inadequate for accessing dental care [6,60,65]. In the US, finding dentists who accept Medicaid (a joint federal and state program that helps cover medical costs for some people with limited income and resources) can be problematic [6]. In particular, orthodontic care is typically not approved except in extreme cases deemed medically necessary rather than aesthetic [65].

In a US study, lack of medical insurance was identified as a barrier to care leavers engaging with dental health services. When controlling for gender and post-care living arrangements, care leavers who did not have dental insurance were 93.5% less likely to have their dental needs met than those who had dental insurance. A lack of support to understand their Medicaid eligibility, how to maintain their eligibility, and the Medicaid application process were barriers to dental care access [17]. Certain US states have identified a number of strategies for ensuring healthcare coverage for youth who age out of foster care [35]. These are collaboration between the child welfare and Medicaid systems; coordinated data systems that automatically notify Medicaid when a young adult is ageing out of foster care; ongoing training for child welfare and Medicaid staff; and education of youth about how to enrol and recertify in their State as well as their potential eligibility. Some states use an automatic enrolment and annual recertification process that requires no or minimal youth involvement, while others use a life-skills approach that requires youth to be proactive and educated about their entitlements and obligations to obtain those entitlements. The automatic enrolment and recertification process yields higher initial healthcare coverage rates for care leavers than the life-skills approach. 

### 3.7. Factors Affecting CYP in Care and Care Leavers’ Dental Experience 

#### 3.7.1. The Additional Needs of CYP in Care and Care Leavers

CYP may enter care because of neglect and abuse; the moving into care itself can also be a traumatic event [76]. Such childhood adverse events can have ongoing consequences for the child, who may express the difficulties they are experiencing via behaviour that is seen as “difficult” or “inappropriate”. The experience of receiving dental care has been shown to trigger past trauma experienced during childhood sexual abuse [77]. Such behaviours and reactions may be challenging for dental health professionals when trying to deliver care [3]. This can be made more difficult because CYP with behavioural issues may also have more unmet dental needs [65]. A diagnosis of ADHD [47] was found to negatively influence the likelihood of CYP in care receiving treatment. 

#### 3.7.2. Meeting the Additional Needs of CYP in Care and Care Leavers

Studies from the UK and Sweden found that building trustful relationships between dental professionals and patients is important to allay the anxiety CYP in care experience around attending the dentist [3,78]. Carers in an Australian study reported that the way dental health professionals interact with children plays a key role in their access to oral health services [7]. Carers identified that CYP with behavioural or emotional difficulties can be challenging to treat and may require additional time from professionals to receive dental care and the creation of a calm environment [3]. 

Providing longer appointments can be a challenge. For example, in the UK, due to the National Health Service contract, general dentists found providing such appointments within the funding restrictions problematic [3]. 

### 3.8. RQ 3: What Pathways Have Been Developed to Improve Access to Oral Health Care for Children and Young People in Care and Care Leavers?

This scoping review search identified six papers/reports related to developing pathways to improve access to oral health for care-experienced children and young people. These were an intervention development study from Australia [79]; three peer-reviewed evaluation studies from the UK and Australia which explored pathways to improving access to dental care for CYP in care [3,67,80]; and a report from the USA [81] describing specialised Medicaid managed care programs for CYP in foster care and a report from the UK. The latter examined a pilot care pathway to oral health advice for looked-after children. This initiative involved training non-dental health professionals involved in the care of looked-after children to provide initial mouth checks and to offer advice as part of the statutory health assessment [82]. Only the US study related to care leavers. Of the six papers/reports, one focused on dental care [3], and the others [67,79,80,81] mentioned dentistry as part of an exploration of CYP’s access to health care in general. 

### 3.9. Referral Processes and Interagency Working

A lack of communication within and between agencies (e.g., social care, medical and dental care) and foster carers can hamper information sharing and engagement between different stakeholders. Fragmented care with frequent placements for children in care exacerbates this problem [80]. All four studies identified the importance of improved inter-agency working and establishing agreed referral processes in facilitating children in care’s access to dental care. Interagency work across dental, medical and social care is valued by the professionals in these fields and foster carers as being a positive influence on dental care access [3,67,80] and credited for facilitating timely access to services [3]. Key components of successful interagency work which were identified were designated responsibility for health advocacy roles [67], centralised referral processes and clear articulation of responsibilities for the monitoring and coordination of referrals [67,79] and the sharing of dental health action plans across agencies [3]. The establishment of good communication channels across agencies enables incidents of non-attendance to be shared and facilitates appropriate follow up. Feeding dental health data into the social care system facilitates improved record keeping, including recording whether statutory demands have been met [3]. A centralised referral process was found to assist carers in understanding professional responsibilities in relation to the monitoring and coordination of referrals [79]. Having a standardised referral form helped to support the referral process and aid common understanding [3,79].

### 3.10. Providing a Designated Pathway to Dental Care 

In the UK, there is no universally adopted designated dental care pathway (DDCP) for CYP in care, and there are significant regional differences in care pathways, non-attendance and other care policies, which can result in geographic inequalities in dental access [53]. A lack of specialist pathways to access care [3] puts the onus on carers to source dental care for the CYP they are caring for. The study by Williams et al. [3] explored the impact of a designated dental care pathway (DDCP) for CYP in care in the UK. Key outcomes of this study included improved interagency integration and support by key professionals and improvements in communication and documentation of dental assessments and outcomes. The dental pathway facilitated dental care access for children entering care, met the dental needs of service users and offered a consistent dental service regardless of the number of placement moves. 

In the USA [81], some states are transitioning children and youth in foster care to specialised Medicaid managed care programs to improve care coordination and healthcare quality, including dental care. In Georgia, children and youth have a care coordinator and receive access to tailored healthcare services for their unique needs, such as clinical trauma screening, wellness visits, and preventative services. This includes dental cleaning twice a year. Both Texas and Wisconsin give children and youth access to a specialised medical home for coordinated care where they have timely access to comprehensive health services, including dental care. Texas offers rewards for accessing preventative services, while Wisconsin provides ongoing health care for a year after leaving foster care.

## 4. Discussion

### 4.1. Statement of Principal Findings

This scoping review highlights the global paucity of data on CYP in care and care leavers’ access to dental care, their experience of dental care and initiatives to facilitate their access to dental care. The studies indicate that CYP, before entering care, in care and after leaving care, experience poorer access to dental health care than their peers despite having higher treatment needs. The available research shows the wide range of factors that impact on the ability of CYP in care and care leavers to access dental care, which spans from the individual and psycho-social to the systems created to meet the dental care needs of this vulnerable group. The availability of dental services and their accessibility in terms of distance and cost impact access, and CYP is reliant on carers and supporters to overcome these barriers. In situations where they are frequently relocated with short-duration placements, the opportunity to have a “home” dentist and continuity of care is significantly diminished. This is particularly relevant given the importance of dental professionals building trusting relationships with CYP who may have experienced adverse childhood events and/or have dental anxiety. Such transience also makes the effective communication and the passing of relevant information between dental professionals, supporting agencies and carers more problematic. Dental professionals can experience difficulties in meeting the needs of CYP in care and care leavers due to time restraints and the challenges that working with anxious and troubled young people can present. Health and social care professionals and carers can lack familiarity with and ability to negotiate the systems for accessing dental health care for the CYP in their care. Various pathways to access have been developed in the UK, but there is a lack of information on pathways operational in other countries and a lack of knowledge of the impact on access. Care leavers are significantly underrepresented in the research evidence in terms of understanding their experiences of dental care, the barriers and facilitators to access and interventions to facilitate access. There is insufficient evidence to explore whether the type of care arrangement (formal/informal foster care, residential care) impacts access to dental care. 

### 4.2. Comparison with Existing Literature

The findings of this scoping review build on those found in previous research, which show that care-experienced CYP, despite having higher levels of oral health needs, have lower levels of dental care access than their peers [46] and experience multifactorial barriers to accessing dental care [40]. There are very few systematic or scoping reviews which address access to dental care by CYP in care or care leavers, and for oral health in general, the evidence base is lacking. A systematic review and meta-analysis by Taylor et al. [83] examined the policies, programmes and interventions that improve outcomes for young people leaving care. This included health outcomes. With the exception of extended care policies, this review was unable to determine whether one approach was better than another due to the poor quality of the evidence base. The systematic review by Mensah et al. [10] evaluated organisational models for the systematic delivery of health and dental care to children and adolescents in out-of-home care. They concluded that the studies available were of insufficient quality to determine the effects of different organisational models for providing health and dental care to these CYP. 

Reviews by Vinnerljung and Jones [84,85] confirm the general lack of evidence on the effectiveness of interventions and strategies for improving access to and meeting the oral health care needs of CYP in care and care leavers. Hurry et al. [40], in their review of dental access pathways for children in care in England, identified four dental care pathway models: care navigation, facilitated access, nurse-led triage and referral, and signposting to local dentists with multi-agency information sharing. The current review highlights the initiatives being taken in the US to facilitate access to preventative and treatment-based oral health care by children in care and care leavers via coordinated managed-care programmes. The review findings are compatible with those of Marcus et al. [86], which highlight the impact of carers’ cultural and linguistic diversity on children’s access to dental care. Both Khalid et al. [87] and Shanthi et al. [88] carried out systematic reviews of the prevalence of dental neglect and the factors associated with such neglect in CYP. They not only highlighted the higher risk of dental neglect in care-experienced CYP but also emphasised the impact of this neglect on the personal and social lives of children and the need for policies to support improved reporting of this kind of neglect by dental professionals.

The findings of this review In relation to dental care access and provision are consistent with studies that have examined the challenges that children in care and care leavers experience in accessing (non-dental) health care. In particular, the challenges arising at the interface between social care and health care systems [89,90], carers lack of access to past medical history [91], placement instability [92] and the impact of past childhood trauma impact on children and young people’s willingness to engage with health professionals has been highlighted [93]. 

### 4.3. Implications for Policy and Practice

Many aspects of access are beyond the control of individual dental care professionals; however, this review highlights the importance of building a relationship with and creating a positive environment for CYP in care and care leavers attending dental care services. With the high levels of adverse childhood events experienced by CYP in care [10], giving all dental health professionals who work with this vulnerable population the opportunity to receive training in trauma-informed dental practice has the potential to facilitate access [94]. 

The studies IIded in this review showed that not all countries have a health assessment as part of a statutory welfare assessment for CYP in care [5,95]. Even where such statutory assessments exist for vulnerable CYP, referrals may not be made to health professionals (including dental services) in a timely manner [96]. The introduction of statutory dental health assessments for CYP in care, regardless of their type of placement, with a strong framework for referral would support greater access (Kling). 

To avoid inequalities in the provision of dental care for CYP in care and care leavers, there needs to be legal clarity about roles and responsibilities, e.g., for consent [97], strong systems and adequate resources to ensure that CYP in the care of authorities are treated fairly with regard to the needs of the individual child. The creation of national and local level guidance on how to create and implement high-quality, effective models of care would help support corporate parents and health and social care professionals to facilitate dental care access.

Stakeholders who are active or interested in facilitating access to dental care by CYP in care and care leavers come from a number of different disciplines, including social, medical and dental care. Cross disciplinary working can be challenging but is necessary if the complex picture of dental care access is to be clarified and new approaches to providing care developed. A first step could be the establishment of a virtual research network of researchers, academics, social, medical and dental care professionals, care-experienced CYP and other stakeholders. This forum would provide the opportunity to share experience and evidence so that challenges and facilitators can be identified and best practices developed. It would also raise the profile of this important issue and give a voice to those people most affected by decisions on systems and pathways: the children and young people themselves.

### 4.4. Unanswered Questions and Future Research

Research on CYP in care and care leavers’ access to dental care is sparse, and what does exist comes almost exclusively from countries categorised as having a very high HDI. The lack of evidence on the access or provision of care in low/medium or high HDI countries is stark. The impact of different care settings and time spent in care on access remains uncertain. Evidence on the pathways provided to facilitate access and what systems work best to meet the needs of CYP in care and care leavers is also lacking. All these are areas where further research is needed. 

The absence of the voice of the children and care and care leavers in the research included in this review is striking. This is consistent with the systematic review by Smales et al. [98], which found that the voices of young people in out-of-home care have been underrepresented in research examining their health and health care needs. Future planning and design of interventions would benefit from including CYP, care leavers and carers to ensure they are meaningful, tailored and fit for purpose. Additionally, there is a need for robust evaluations to focus both on outcome and process measures to deepen understanding of the experiences of CYP in care/carers and to develop strategies to improve access for their diverse and complex needs. 

### 4.5. Strengths and Limitations

This scoping review is the first to synthesise the global literature relating to access to dental care for children in care. The review was conducted systematically using rigorous methods. To reduce the risk of selection and publication bias, more than one reviewer was involved in study selection, and grey literature sources were included. 

The review includes studies from across the world, from a range of different geographical and care settings using a variety of research methods. This is a strength but also a limitation of the review as the heterogeneity of these characteristics plus differences in CYP’s age, service provision, and statutory guidelines means that interpretation of results must be made with caution. 

The aim of a scoping Ieview is to provide an overview of the evidence, and generally, an assessment of the quality of the evidence is not performed [99]. This is a limitation of the review as individual studies in the review may include biases in their results or conclusions. 

Given that activity and evaluations, especially in relation to pathway development, may not be published in peer-reviewed journals or grey literature, they would be missed by the search strategy employed in this scoping review. In the recent scoping review by Hurry [40], they expanded their investigation to include information gleaned from personal contacts working in the field. This enriched their findings but, given the global reach of the present review, was not a feasible option. 

## 5. Conclusions

This global review of the research evidence on CYP in care and care leavers’ access to dental care and the factors influencing such access demonstrates that this vulnerable population are not receiving the same standard of care and is disadvantaged in their access to dental health services. Organisational, psycho-social and logistical factors influence their ability to access services, and although they are a group experiencing high treatment needs, there are significant barriers to their needs being met. 

There are promising initiatives, at national and local levels, to develop pathways to help CYP, carers and social care professionals to navigate systems via signposting and multi-agency working. However, these need more rigorous evaluation and evidence is needed to identify effective approaches within different health and social systems and legislative environments. The sharing of research evidence, practice and evaluation data among all significant stakeholders may help highlight the inequalities in dental access experienced by these CYP and facilitate multi-disciplinary work toward reducing them. 

## Data Availability

The data that supports the findings of this study are available in the Supplementary Material of this article in the data extraction table.

## Supplementary Materials

The following supporting information can be downloaded at: https://www.mdpi.com/article/10.3390/dj12020037/s1. Table S1: Data Extraction Table.

## Appendix A

**Table A1:** Search Strategy.

1	(child* or youth* or adolescen* or teen* or young people).tw.
2	care leaver*.tw.
3	leaving care.tw.
4	(transit* adj3 (care or services)).tw.
5	Child* in care.tw.
6	looked after child*.tw.
7	accommodated child.tw.
8	(“out of home care” or “out of home placement”).tw.
9	kinship care.tw.
10	((adoption or adopted) adj3 child*).tw.
11	adopted child/
12	(custod* adj3 care).tw.
13	Orphan*tw.
14	(placement adj3 care).tw.
15	public care.tw.
16	(foster adj1 (care* or home* or family or parent*)).tw.
17	(institutional* adj3 (care or home*)).tw.
18	(group adj1 home*).tw.
19	(residential adj3 (care or home* or facilit*)).tw.
20	(welfare adj3 (care or system*)).tw.
21	statutory care.tw.
22	(care ajd3 local authority).tw.
23	care order*tw.
24	(substitute adj1 (care or famil*)).tw.
25	special guardian*.tw.
26	Kafalah.tw.
27	(unaccompanied adj3 asylum).tw.
28	(unaccompanied adj3 refugee*).tw.
29	(“Children Act 1989” or “Children Northern Ireland Order 1995” or “Children Scotland Act 1995”).tw.
30	or/5–29
31	dental health/
32	dental procedure/
33	exp tooth disease/
34	exp dentist/
35	(oral adj3 (health* or hygiene or care)).ab,kw,ti.
36	dental.ab,kw,ti.
37	((tooth adj3 (health* or hygiene or care or brush* or floss*)) or toothbrush*).ab,kw,ti.
38	(teeth adj3 (health* or hygiene or care or brush* or floss*)).ab,kw,ti.
39	dentist*.ab,kw,ti.
40	or/31–39
41	(1 or 2 or 3) and 30 and 40

* search strategy adapted for each database <1974 to 2 January 2024>.
